# Relevance of Chronic Total Occlusion for Outcome of Ventricular Tachycardia Ablation in Ischemic Cardiomyopathy

**DOI:** 10.1155/2022/6829725

**Published:** 2022-07-18

**Authors:** Julia Anna Lurz, Eileen Schmidt, Karl-Patrik Kresoja, Federica Torri, Sebastian König, Angeliki Darma, Arash Arya, Livio Bertagnolli, Gerhard Hindricks, Borislav Dinov

**Affiliations:** ^1^Department of Electrophysiology, Herzzentrum Leipzig at University, Leipzig, Germany; ^2^Department of Cardiology, Herzzentrum Leipzig at University, Leipzig, Germany

## Abstract

**Background:**

Catheter ablation of ventricular tachycardia (VT) in patients with ischemic cardiomyopathy (ICM) is an effective tool to prevent VT recurrences. Chronic total occlusion (CTO) represents a clinically relevant entity in ICM patients and is an independent predictor of ventricular arrhythmia and mortality. The effects of CTO on the outcome of VT ablation are not well-studied.

**Objective:**

This analysis aimed to identify the impact of CTO, revascularized, or not revascularized, on the outcome of VT ablation.

**Methods and Results:**

Of 385 consecutive subjects with ICM-VT who underwent catheter VT ablation for monomorphic VT at Heart Center Leipzig between 2008 and 2017, 108 patients without CTO and 191 patients with CTO were included in the analysis. Within a median follow-up time of 557 days (IQR 149, 1095), VT recurred in 77 (40%) patients in the CTO and 40 (37.0%) in the non-CTO cohort (*p* = 0.62). In a multivariable model, a 10% stepwise change in LVEF as well as ICD on admission was associated with VT recurrence (HR_adj_ 1.82, 95% CI 1.04–3.18 and HR_adj_ 1.35, 95% CI 1.23–1.61, respectively). Of the CTO cohort before ablation, 45% had received revascularization, which was independently associated with a higher risk for VT recurrence (HR 2.12, 95% CI 1.35–3.34) as compared to nonrevascularized CTO.

**Conclusion:**

In ICM patients with and without CTO, VT ablation was associated with equal effectiveness with regard to VT recurrence. However, in revascularized CTO patients, the risk of recurrence of VT after ablation was significantly increased.

## 1. Introduction

Catheter ablation of ventricular tachycardia (VT) is an established therapy in patients with ischemic cardiomyopathy (ICM) [1, 2]. VT recurrences after ablation are common, leading to appropriate implantable cardioverter-defibrillator (ICD) therapy in 43% within the first year [3]. Further risk stratification of patients with a high probability of VT recurrences could guide individual therapeutic decision-making.

Chronic total occlusion (CTO) represents a clinically relevant entity as it is a frequent finding in patients with ICM affecting up to 56% of patients undergoing coronary angiography and as much as 69% of patients receiving an ICD [4, 5]. A meta-analysis of patients with and without ICDs concluded that CTO of an infarct-related artery (IRA) was associated with a 2-fold higher risk for ventricular arrhythmia (VA) and 1.7-fold higher all-cause mortality [5]. Di Marco et al. suggested IRA-CTO as an independent predictor of VT recurrence after ablation [6]. However, the implications for patients undergoing VT ablation remain understudied [4–8]. Furthermore, recent evidence from case reports and observational studies suggested possible proarrhythmic effects of CTO revascularization in patients with the substrate for VA [9, 10]. This analysis aimed to evaluate the role of CTO as a predictor for VA recurrence after ablation and to determine the impact of successful revascularization procedures, surgical, or interventional on outcome after VT ablation.

## 2. Methods

### 2.1. Patient Cohort and Definition of Baseline Characteristics

This observational study assessed the outcome of first-time catheter ablation of VT in patients with prior myocardial infarction (MI) according to the presence or absence of CTO in a high-volume tertiary center between 2008 and 2017. It was approved by the local ethics committee and all patients gave written informed consent for the procedure. The study cohort and the investigation conform with the principles of the Declaration of Helsinki.

Prior MI was determined by past medical history, local wall motion abnormalities in transthoracic echocardiography, or *q*-waves in 12-lead electrocardiogram (ECG). All patients were categorized into two groups: (1) CTO: defined as infarct-related (IRA-CTO) if the occluded vessel was in the territory of prior MI, transmural scar, or pathological *q*-waves on ECG, or non-infarct-related (non-IRA-CTO) if the CTO affected a nonculprit coronary artery and (2) non-CTO. CTO was defined as chronic total occlusion of the coronary artery for an estimated duration of at least 3 months resulting in TIMI flow 0 [11].

The study cohort was further analyzed according to the revascularization status. Patients with successful interventional revascularization or patent coronary artery bypass graft (CABG) supplying the CTO were considered revascularized CTO. Unattempted or unsuccessful revascularization procedures or occluded CABG was considered non-revascularized. All revascularization procedures were performed before VT ablation.

### 2.2. Ablation Procedure

The decision for VT ablation was based on recommendations of the HRS/EHRA task force guidelines. Patients with recurrent sustained VTs, requiring ICD therapies with or without amiodarone were scheduled for catheter ablation. VTs with reversible causes as well as idiopathic VTs were excluded. The ablation procedure was performed according to the local standards of care under deep sedation. Programmed ventricular stimulation in the right ventricular apex (and outflow tract or left ventricle if not inducible otherwise) was performed at the beginning using four different basic cycle lengths (CL) (500, 430, 370, and 330 ms) and up to 3 extra stimuli until ventricular effective refractory period or coupling interval of 200 ms was reached. The signals were recorded by a multichannel recording system (Prucka CardioLab; GE Healthcare, Waukesha, WI, USA). The morphology of induced VT was compared with that of the spontaneous VT and was considered as clinical VT in case of QRS morphology or CL match with previous ECG recordings or ICD EGMs. We used fluoroscopy-guided single transseptal puncture and a long steerable sheath (Agilis, Abbott, St. Paul, MN, USA) to access the LV. In suspected epicardial VT, a subxiphoid puncture using a shorter steerable sheath (Agilis EPI, Abbott, St Paul, MN, USA) was performed before the transseptal puncture. Intravenous heparin was administered before the transseptal access to maintain an activated clotting time of 250–300 s.

Electroanatomic mapping (EAM) of the LV was performed using the EAM mapping system (Carto XP and 3; Biosense-Webster, Diamond Bar, CA, USA). The substrate was defined as low-voltage areas (0.5–1.5 mv) and abnormal fragmented or delayed electrograms. Where possible, activation mapping was performed to delineate the reentry circuit. Clinical and all sustained monomorphic VT with a CL slower than 250 ms were targeted. Radiofrequency alternating current was delivered by irrigated tip ablation catheter (Thermocool, Biosense Webster, Diamond Bar, CA, USA) in a power-controlled mode (50 Watts, flow rate 30 ml/min). EPS according to the above-mentioned protocol was used to test for post-ablation inducibility. The ablation was defined as a complete success if non-inducibility of any sustained monomorphic VT was achieved; and partially successful if only the clinical VT was ablated.

### 2.3. ICD Programming

The following aspects were considered for ICD programming: complete elimination of all inducible VTs, the CL and the hemodynamic relevance of inducible VTs at the end of the procedure, and whether VT could be terminated by overdrive stimulation. If VTs were still inducible, three zones were programmed following the rule of 20 ms above CL of the slowest VT or 50 ms in case of amiodarone treatment. The fast VT/VF zone was set up at 260 ms.

### 2.4. Follow-Up

The primary outcome was VA recurrence defined as adequate ICD therapy delivery for sustained VT, ventricular fibrillation (VF), or VT below ICD detection. Time-to-first VA recurrence after catheter ablation was identified by ICD interrogations and from medical records.

### 2.5. Statistical Analysis

Data for continuous variables were presented as mean ± standard deviation (SD) if they follow parametric distribution or as median and interquartile range (IQR) if nonparametrically distributed. Distribution was tested using Kolmogorov–Smirnov test. Categorical variables were presented as frequencies and percentages and compared with Fisher's exact test. Continuous variables were compared using either a Student's *t*-test or Mann–Whitney *U* test, as appropriate. Cox regression analysis identified predictors of the primary outcome and adjusted for baseline differences between CTO and no CTO patients. In the multivariable Cox regression analysis characteristics that exhibited significant differences between the groups with *p* < 0.1 were included. A two-tailed *p* < 0.05 was considered significant. All data were analyzed using SPSS Version 20 (IBM, Armonk, NY, USA).

## 3. Results

### 3.1. Patient Cohort

**Figure 1:**
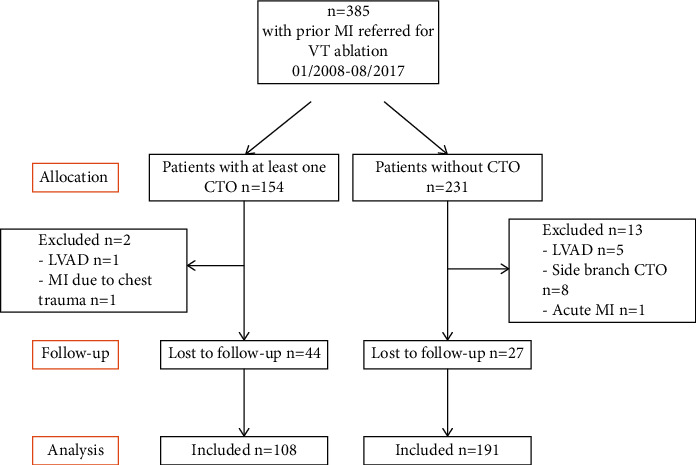
Study flow chart. CTO = chronic total occlusion; LVAD = left ventricular assist device.

Of 385 consecutive subjects with ICM-VT who underwent catheter VT ablation for monomorphic VT between 2008–2017, 108 patients without CTO and 191 patients with CTO were included in the analysis. The median follow-up time was 557 days (IQR 149, 1113). The selection of the patients with respect to inclusion, exclusion criteria, and follow-up is schematically outlined in Figure 1.

### 3.2. Baseline Characteristics

Baseline characteristics of CTO and non-CTO patients as well as of the subgroups of non-revascularized and revascularized CTO patients are shown in Table 1. CTO patients (*n* = 191) were older, had more severe coronary artery disease (CAD), more often ICDs on admission, poorer left ventricular ejection fraction (LVEF), and received more frequently amiodarone as compared to non-CTO patients (*n* = 108). The female gender was considerably underrepresented in both groups, but there were even fewer female patients in the CTO group. At discharge, there was no difference between the groups with respect to ICD and amiodarone therapy. Notably, the time from MI to ablation did not differ between groups. Within the CTO group, revascularized patients more frequently exhibited arterial hypertension, CABG, multivessel disease, and CTO of the left anterior descending artery as compared to non-revascularized patients.

### 3.3. Outcome Data

VT recurred in 117 patients, in 77 of the CTO (40.3%), and 40 (37.0%) of the non-CTO cohort (Table 2). Follow-up did not differ between the non-CTO and CTO groups, and ablation of clinical VT was achieved in 81% and 79%, respectively. The epicardial approach was more frequently applied in non-CTO patients (6 vs. 2%; *p*=0.001). Overall, 13 patients suffered from major ablation-related complications without differences between the groups. Persistent inducibility of VT at the end of ablation was associated with VA recurrence; hazard ratio (HR) = 1.78; 95% confidence interval (CI), 1.19–2.65; *p*=0.005.

### 3.4. CTO Effects on VT Recurrence and All-Cause Mortality

**Figure 2:**
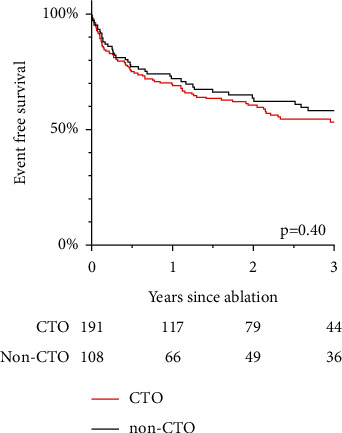
Recurrence of VT in chronic total occlusion (CTO) and non-CTO patients.

The presence of a CTO was not associated with an increased risk for the primary outcome in univariable analysis: HR 1.14, 95% CI 0.77–1.69; *p*=0.40 (Figure 2). This also applied after adjustment for the baseline variables that were different between the groups (age, sex, multivessel CAD, ICD on admission, and LVEF) : HR_adj_ 1.23, 95% CI 0.82–1.85; *p*=0.32. In the multivariable model, a 10% stepwise decrease in LVEF and ICD on admission were the only variables associated with the primary outcome (HR_adj_ 1.82, 95% CI 1.04–3.18; *p*=0.001 and HR_adj_ 1.35, 95% CI 1.23–1.61; *p*=0.023, respectively).

**Figure 3:**
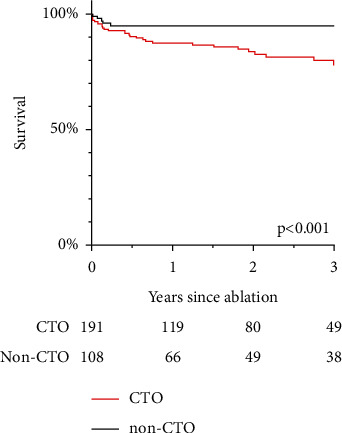
Survival curve of chronic total occlusion (CTO) and non-CTO patients.

With respect to mortality, non-CTO patients had a lower hazard ratio for the occurrence of all-cause mortality in univariable analysis (HR: 0.30, 95% CI 0.12–0.78; *p* < 0.001; Figure 3). This did not remain significant after adjustment for the covariables mentioned above: HR_adj_ 0.48, 95% CI 0.18–1.28; *p*=0.13. Again, a 10% change in LVEF was associated with all-cause mortality after multivariable adjustment (HR_adj_ 1.48, 95% CI 1.04–2.11; *p*=0.034).

### 3.5. Effects of Revascularization Status and Localization of CTO

**Figure 4:**
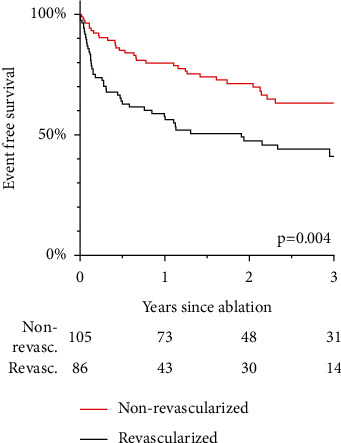
Recurrence of VT in revascularized vs. nonrevascularized patients.

Among CTO patients, 86 (45%) had undergone successful revascularization procedure prior to VT ablation. Of those, 69 (80,2%) had undergone CABG and 17 (19.8%) interventional revascularization procedure. Successful revascularization was associated with a higher risk for VT recurrence: HR 2.12, 95% CI 1.35–3.34; *p* = 0.004 (Figure 4). Localization of the CTO-affected vessel was not associated with unfavorable outcomes (*p* = 0.25). Revascularization, however, remained associated with an increased probability for the primary outcome (HR_adj_ 2.36, 95% CI 1.38–4.04; *p* = 0.002) after adjustment for the baseline confounders arterial hypertension, multivessel-CAD, presence of CABG and CTO of the left anterior descending artery. These results remained stable in a subanalysis of 97 unrevascularized and 82 revascularized patients with myocardial infarction to ablation interval of greater than 180 days: HR 2.17, 95% CI 1.3–3.45; *p* = 0.001). There was no difference whether the CTO was collateralized (*p* = 0.40), and revascularization was not associated with all-cause mortality (HR 1.70, 95% CI 0.81–3.56; *p* = 0.16).

### 3.6. Predictors of VT Recurrence in Non-CTO Patients

In non-CTO patients, LVEF was predictive for VT recurrence in the multivariable model (Table 3).

### 3.7. Effects of IRA and Non-IRA

**Figure 5:**
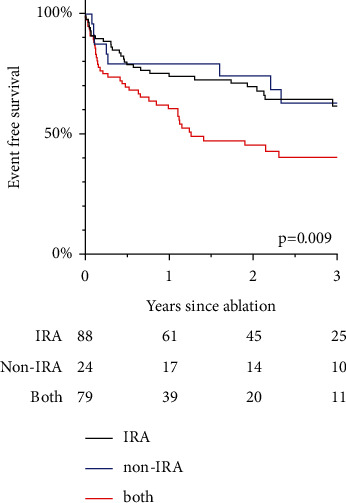
VT recurrence comparing infarct-related artery (IRA), non-IRA and presence of both.

Examining the influence of the CTO affected vessel patients were divided into three groups: IRA-CTO only, IRA + non-IRA-CTO, non-IRA-CTO only. Univariable logistic regression analysis revealed a higher VT recurrence rate for patients with both IRA- and non-IRA-CTO as compared to IRA-CTO only (Figure 5): HR 1.96, 95% CI 1.21, 3.16; *p*=0.009. There was no difference in outcomes between IRA-CTO only and non-IRA-CTO only (*p*=0.96).

## 4. Discussion

The main findings of this study are as follows:

1. Long-term VA recurrence after ablation was comparable in patients with and without CTO despite more severe baseline characteristics of the former.
2. The presence of a CTO was associated with a higher risk of death in the univariable analysis.
3. Remarkably, revascularization of CTO was associated with a more unfavorable outcome after VT ablation.

### 4.1. CTO and Non-CTO

In patients with ICM, CTO of at least one coronary artery is a common finding affecting up to 62%. [4, 5]. Several retrospective studies analyzed the impact of CTO on the occurrence of VA and mortality in ICM patients indicating worse outcomes if a CTO was present [7, 8, 12–15]. In the context of catheter ablation for VT, Di Marco and colleagues reported on the importance of CTO for the outcome of VT ablation [6, 16].

In patients with transmural myocardial infarction in multivessel-CAD, CTO of a non-IRA was associated with early and late mortality, but the driver appeared to be the continuous deterioration of LVEF [7, 13, 14]. In patients presenting for ICD implantation, those with CTO had a higher risk for VA recurrence and mortality, but the CTO population was sicker exhibiting more risk factors such as atrial fibrillation, multivessel-CAD, and lower LVEF [15, 17, 18]. Interestingly, the collateral flow was associated with a trend toward a higher VT recurrence rate [17]. Nonetheless, Raja et al. did not find an association between VA, death, and CTO in patients with ICD over a median FU period of 4.1 years [4]. A recent meta-analysis, however, concluded that CTO was associated with a higher risk of VA or appropriate ICD therapies (HR 1.99, 95% CI 1.5–2.6) but not with higher mortality. Yet, when CTO affected an IRA, this was predictive for all-cause (but not cardiac) mortality [5]. A major issue in the CTO research is the significant heterogeneity of the data, making the comparison a difficult task.

To our knowledge, the only study that analyzed outcomes after VT ablation suggested that the CTO of an IRA was a strong predictor for VT recurrence with a 4-fold higher risk of new VT. Furthermore, the EAM showed a larger, more heterogenous, and arrhythmogenic substrate in the CTO cohort. This study had several limitations such as the small heterogeneous control group consisting of patients without any CTO or CTO of a non-IRA. A major difference from our study was that revascularized CTOs were not considered CTO [6].

In this much bigger cohort, we could not find an association between the presence of CTO and VA recurrence after catheter ablation. Although the CTO group consisted of older and sicker patients, VT ablation successfully suppressed VA in both groups alike. During the long-term follow-up, VA-free survival was achieved in 59.7% in the CTO- and 63.0% in the non-CTO group. In contrast to previous studies suggesting a higher risk of VA recurrence when an IRA-CTO was present, we found that IRA-CTO was not associated with the risk of unfavorable outcomes. However, the presence of both, IRA- and non-IRA-CTO, in one patient showed a higher risk for VA recurrence. In our opinion, this was linked to a higher “CTO-burden” rather than to the affection of the IRA.

In line with previous data, all-cause mortality in CTO patients was higher, but this effect was mitigated after adjustment for LVEF, age, previous CABG, presence of ICD, and multivessel disease. Altogether, the results imply that the unfavorable outcomes after VT ablation in patients with CTO were more likely related to the natural progression of heart failure than to the properties of the arrhythmogenic substrate or the quality of ablation itself. This emphasizes once more the essential role of heart failure therapy as a part of the treatment of ischemic VT irrespective of ablation outcomes.

### 4.2. Revascularization of CTO and Pathophysiological Considerations

The primary indication for the revascularization procedure of a CTO is symptom improvement or salvage of viable hibernating myocardium. Whether it also leads to improvement of other cardiovascular outcomes, such as LVEF, VA, and mortality remains controversial [4, 19]. A meta-analysis revealed a mortality benefit of revascularization only for patients with viable myocardium [20]. Recently, an observational study described new-onset monomorphic VTs in previously stable patients shortly after successful revascularization of CTO and suggested possible proarrhythmic effects of restoring the blood supply in areas with CTO. In line with previous data, restoring the coronary flow in an IRA-CTO was associated with a risk for VT occurrences [10]. So far, there are no data on the influence of revascularization status on the recurrence of VT after catheter ablation in patients with ICM-VT.

It is believed that CTO revascularization can salvage hibernating myocardium, prevent further deterioration of LVEF, and possibly restore the electrical stability of the myocardium. However, we could not observe an improvement in survival or VT occurrence after CTO revascularization before VT ablation. On the contrary, CTO revascularization was independently associated with a 2-fold increased risk for VA recurrence. The assumption that the poorer outcomes in surgically revascularized patients were a result of difficulties with the epicardial access was disproved by the similar attempts for epicardial access in both subgroups.

Some data have suggested a proarrhythmic effect of blood flow restoration in areas of chronic hibernating myocardium as chronic ischemia may cause heterogeneity in innervation [21]. Inhomogeneity in sympathetic innervation assessed by C (11)-labeled hydroxyephedrin in patients with hibernating myocardium persisted after revascularization despite restoration of myocardial contractility [22]. Accordingly, in the PAREPET trial sympathetic denervation predicted death from sudden cardiac arrhythmia (TCL < 250 ms) in patients with ICM irrespective of LVEF and infarct volume [23].

These observations suggest that revascularization of a CTO may leave viable but denervated myocardium exhibiting denervation hypersensitivity, which in turn may increase the susceptibility to ventricular arrhythmias. Revascularization may also cause an increase of the Purkinje fiber automaticity or induce afterdepolarizations leading to focal VA [24]. However, whether sudden cardiac arrhythmic death varies between revascularized and nonrevascularized patients is subject to larger and prospective studies. Ultimately, the benefits of CTO revascularization should be carefully weighed against the risk of an increase in VA burden. Certainly, the progressive nature of heart failure may influence the time and burden of VA recurrence [25].

### 4.3. Limitations

This is a retrospective observational study and has the limitations typical for all retrospective studies such as selection bias, incomplete follow-up, and confounding resulting from differences in the groups. A specific limitation is the lack of data regarding the timing between the CTO revascularization and VT ablation. Only a minority of patients received CMR before VT ablation, which precluded a detailed analysis of the scar. During 2008–2017, CMR was not routinely performed in patients with ICDs because of missing data on CMR safety, CMR-incompatible ICD devices, and lack of dedicated software to analyze the properties of the scar. Additionally, the underlying mechanisms leading to a higher rate of recurrences in revascularized patients remain speculative and might be subject to bias.

## 5. Conclusion

Chronic total occlusion was a frequent finding in patients with ICM-VT requiring catheter ablation. Even though CTO patients had more advanced disease progression, the acute and long-term outcomes of catheter ablation were good and comparable to those of non-CTO patients. CTO revascularization before ablation was associated with a higher risk for recurrence of VA after ablation. CTO revascularization before VT ablation should not be pursued just to improve clinical outcomes after ablation.

## Figures and Tables

**Table 1:** Baseline parameters for CTO and non-CTO as well as nonrevascularized and revascularized patients.

*Baseline characteristics*	All Patients	Non-CTO	CTO	*p* value	Non-Revascularized CTO	Revascularized CTO	*p* value
	*n* = 299	*n* = 108	*n* = 191		*n* = 105	*n* = 86	
Age (years)	69 (60, 76)	65 (57, 73)	70 (62, 77)	0.001	70 (62, 76)	69 (61, 77)	0.58
Female sex	23 (8)	14 (13)	9 (5)	0.01	4 (4)	5 (6)	0.49

*Risk factors*							
Body-mass index	28 (25, 31)	26 (23, 32)	28 (25, 31)	0.48	28 (25, 31)	29 (25, 32)	0.300
Diabetes	105 (35)	33 (31)	72 (38)	0.21	39 (37)	33 (38)	0.77
Hypertension	269 (90)	97 (90)	172 (90)	0.95	90 (86)	82 (95)	0.03
Renal dysfunction (GFR ≤ 60 ml/min)	144 (48)	45 (42)	99 (52)	0.09	50 (48)	49 (57)	0.15

*Coronary arteries*							
CAD				<0.001			0.013
1-vessel	79 (26)	51 (47)	28 (15)		22 (21)	6 (7)	
2-vessel	84 (28)	31 (29)	53 (28)		31 (29)	26 (26)	
3-vessel	136 (46)	26 (24)	110 (58)		53 (50)	67 (67)	
Multi vessel CAD	220 (74)	57 (53)	163 (85)	<0.001	82 (78)	81 (94)	0.002
Previous CABG	124 (42)	19 (18)	105 (55)	<0.001	31 (30)	74 (86)	<0.001
Myocardial infarction affected artery				0.899			0.23
LAD	129 (43)	48 (44)	81 (42)		43 (41)	37 (44)	
CX	22 (7)	7 (7)	15 (8)		8 (8)	7 (8)	
RCA	131 (44)	48 (44)	83 (43)		51 (48)	32 (38)	
>1 vessel	17 (6)	5 (5)	12 (6)		4 (4)	9 (11)	
Myocardial infarction to ablation (months)	474 ± 470	465 ± 606	479 ± 370	0.81	483 ± 425	475 ± 295	0.893

*Characteristics on admission*							
NYHA class III or IV	119 (40)	37 (34)	82 (43)	0.14	47 (45)	35 (41)	0.76
ICD on admission	232 (78)	73 (68)	159 (83)	0.002	86 (82)	73 (85)	0.70
ICD at discharge	283 (95)	101 (94)	182 (95)	0.39	102 (97)	80 (93)	0.17
Betablocker	279 (93)	102 (94)	177 (93)	0.56	97 (91)	81 (94)	0.49
Amiodarone on admission	106 (36)	27 (26)	79 (41)	0.006	43 (41)	36 (42)	0.90
Amiodarone at discharge	107 (36)	35 (32)	72 (38)	0.271	40 (38)	32 (38)	0.534
VT cycle length (ms)	335 (290, 390)	320 (260, 376)	340 (296, 394)	0.061	355 (315, 400)	335 (290, 400)	0.16
Electrical storm	189 (63)	70 (65)	119 (62)	0.67	64 (61)	55 (64)	0.75
ICD shock	161 (63)	48 (57)	113 (65)	0.20	57 (54)	56 (65)	0.11

*Echocardiography*							
LVEF (%)	33 ± 12	35 ± 12	32 ± 11	0.030	33 ± 10	32 ± 11	0.97
LVEDD (mm)	62 ± 10	61 ± 11	62 ± 9	0.74	62 ± 10	62 ± 9	0.85

*Chronic total occlusion*							0.012
LAD	na	na	67 (35)	na	28 (26)	39 (46)	
CX	na	na	31 (16)	na	19 (18)	12 (14)	
RCA	na	na	93 (49)	na	59 (56)	34 (40)	
Multivessel CTO	na	na	79 (41)	na	31 (29)	48 (56)	<0.001
IRA	na	na	167 (87)	na	91 (87)	76 (88)	0.52
non-IRA	na	na	103 (54)	na	52 (50)	51 (59)	0.25

Values presented as *n* (%), mean ± SD or median (IQR); chronic total occlusion = CTO; circumflex artery = CX; coronary artery disease = CAD; coronary artery bypass graft = CABG; glomerular filtration rate = GFR; implantable cardioverter-defibrillator = ICD; infarct-related artery = IRA; left anterior descending artery = LAD; LV end-diastolic dimension = LVEDD; left ventricular (LV) ejection fraction = EF; New York heart association = NYHA; right anterior descending = RCA; ventricular tachycardia = VT.

**Table 2:** Outcome parameters for CTO and non-CTO patients as well as nonrevascularized and revascularized patients.

	All patients	Non-CTO	CTO	*p* value	Non-revascularized	Revascularized	*p* value
	*n* = 299	*n* = 108	*n* = 191		*n* = 105	*n* = 86	
*Ablation data*							
Ablation of all VTs	210 (70)	79 (73)	131 (66)	0.56	73 (70)	58 (67)	0.53
Ablation of clinical VT	239 (80)	87 (81)	152 (79)	0.83	82 (78)	70 (81)	0.85
No of induced VTs	2 (1, 3)	2 (1, 3)	2 (1, 3)	0.50	2 (1, 3)	2 (1, 3)	0.87
Inducibility post	63 (21)	21 (19)	42 (22)	0.60	22 (21)	20 (23)	0.91
Epicardial approach	8 (3)	6 (6)	2 (1)	0.001	4 (4)	5 (6)	0.21
Epicardial origin	52 (17)	20 (19)	32 (17)	0.75	17 (16)	17 (20)	0.40
Major complication	13 (4)	3 (3)	10 (5)	0.32	6 (6)	5 (5)	0.77

*Follow-up*							
Time to follow-up (days)	557 (149, 1113)	567 (156, 1383)	534 (122, 1055)	0.23	661 (232, 1410)	364 (49, 939)	0.004
Recurrence rate	117 (39)	40 (37)	77 (40)	0.62	32 (31)	45 (52)	0.002
Recurrent VT-CL (ms)	365 (302, 428)	340 (293, 425)	380 (313, 428)	0.26	375 (303, 419)	380 (318, 435)	0.50
Time to recurrence (days)	149 (41, 407)	131 (46, 421)	149 (40, 406)	0.84	211 (63, 503)	94 (29, 337)	0.050
Death in hospital	15 (5)	5 (5)	10 (5)	0.82	4 (4)	5 (6)	0.31
Death at follow-up	34 (11)	5 (5)	29 (15)	0.006	14 (13)	15 (17)	0.17
Reablation during follow-up	51 (17)	21 (19)	30 (16)	0.41	13 (12)	17 (20)	0.16

Values presented as *n* (%), mean ± SD or median (IQR), chronic total occlusion = CTO; cycle length = CL; ventricular tachycardia = VT.

**Table 3:** Outcome predictors non-CTO patients.

	Univariable HR (95% CI)	*p* value	Multivariable HR (95% CI)	*p* value
Age		0.16		
Female sex	0.63 (0.23–1.78)	0.36		
BMI	1.10 (0.80–1.50)	0.55		
Diabetes mellitus	0.62 (0.29–1.30)	0.62		
Hypertension	2.64 (0.64–10.96)	0.12		
Renal dysfunction (GFR ≤ 60 ml/min)	1.91 (1.02–3.56)	0.043		
Multivessel disease	0.82 (0.44–1.53)	0.54		
Coronary artery bypass graft	0.58 (0.23–1.48)	0.25		
NYHA III or IV	2.27 (1.21–4.24)	0.10		
ICD on admission	0.45 (0.21–0.97)	0.041		
Amiodarone on admission	1.20 (0.57–2.52)	0.64		
Ventricular tachycardia cycle length	1.00 (0.99–1.01)	0.67		
LVEF per 10%	0.96 (0.52–0.92)	0.006	0.52–0.92	0.006
LV end-diastolic diameter	1.00 (0.98–1.03)	0.79		

Confidence interval = CI; hazard ratio = HR; implantable cardioverter-defibrillator (ICD); glomerular filtration rate = GFR; left ventricular ejection fraction = LVEF; New York Heart Association = NYHA.

## Data Availability

Access to data is restricted due to patient protection of data privacy.
